# Botanical Drugs as an Emerging Strategy in Inflammatory Bowel Disease: A Review

**DOI:** 10.1155/2015/179616

**Published:** 2015-10-20

**Authors:** Francesca Algieri, Alba Rodriguez-Nogales, M. Elena Rodriguez-Cabezas, Severiano Risco, M. Angeles Ocete, Julio Galvez

**Affiliations:** CIBER-EHD, Department of Pharmacology, ibs.GRANADA, Center for Biomedical Research (CIBM), University of Granada, Avenida del Conocimiento s/n, Armilla, 18016 Granada, Spain

## Abstract

Crohn's disease and ulcerative colitis are the two most common categories of inflammatory bowel disease (IBD), which are characterized by chronic inflammation of the intestine that comprises the patients' life quality and requires sustained pharmacological and surgical treatments. Since their aetiology is not completely understood, nonfully efficient drugs have been developed and those that show effectiveness are not devoid of quite important adverse effects that impair their long-term use. Therefore, many patients try with some botanical drugs, which are safe and efficient after many years of use. However, it is necessary to properly evaluate these therapies to consider a new strategy for human IBD. In this report we have reviewed the main botanical drugs that have been assessed in clinical trials in human IBD and the mechanisms and the active compounds proposed for their beneficial effects.

## 1. Introduction

Inflammatory bowel disease (IBD) is a chronic gastrointestinal inflammatory disorder characterized by alternating relapses and remissions. The two most common types of IBD are Crohn's disease (CD) and ulcerative colitis (UC), which are characterized by exacerbated uncontrolled intestinal inflammation that contributes to worsening of the life quality of the patients and require prolonged medical and/or surgical interventions. The inflammation associated with CD can discontinuously affect all the gastrointestinal tract, from the mouth to the anus, but it is more often localized to the distal small bowel and/or colon. Samples of inflamed bowel obtained from patients with active CD show transmural inflammation with an important accumulation of acute and chronic inflammatory cells within the mucosa, submucosa, and muscularis propia. On the other hand, UC is characterized by a nontransmural inflammation, just localized within the rectum and the large bowel. Typically, the inflammation is restricted to the mucosa and submucosa, with cryptitis and crypt abscesses, although the inflammatory cell composition is similar to CD. The clinical presentation in these intestinal conditions mostly depends on disease location and is characterized by diarrhoea, abdominal pain, fever, bowel obstruction, passage of blood, and/or mucus [1, 2]. Unfortunately, the aetiology of IBD is not fully understood [3], although there is a general agreement that IBD is the result of a complex combination of four main factors: multiple genetic variations, alterations in the composition of the intestinal microbiota, changes in the surrounding environment, and overreactivity of the intestinal mucosal immune response [4]. Thus, genetically susceptible patients build up an exaggerated and uncontrolled immune response in the gastrointestinal tract towards an altered intestinal microbiota that turns into a chronic intestinal inflammation. Similarly to other inflammatory conditions, a broad spectrum of inflammatory mediators is responsible of the pathophysiology of IBD, including cytokines, chemokines, leukotrienes, and prostaglandins, together with reactive oxygen and nitrogen species. Their synthesis and release are severely altered, which participate in the different phases of the inflammatory process that take place in the gut [5].

Considering all the above, IBD treatment pursues two clear goals: firstly, to promote the symptom remission during the acute flare, and secondly, to maintain the remission and control the chronic inflammation to prevent or hold up the reactivation of the intestinal inflammatory process. It is evident that suppression of the exaggerated immune response is crucial for the management of IBD patients. Actually, this is the major aim of the pharmacological therapy, which includes aminosalicylates (sulfasalazine or mesalamine), immunosuppressants (glucocorticoids, azathioprine, methotrexate, and cyclosporine A), and biologicals (infliximab or adalimumab) [6]. Nevertheless, despite the efficacy shown by these drugs, the important rate of side effects may even limit their necessary long-term use [7]. Therefore, the development of new therapies that combine efficacy and safety in human IBD therapy is needed.

In this regard, the use of alternative therapies has emerged as a common approach in gastrointestinal diseases [8]; actually, a study described that almost half of IBD patients have ever taken or currently use complementary remedies [9]. Different factors may contribute to this situation, including the lack of a complete response to standard therapy and the general feeling about a better safety profile of traditional remedies, in combination with the appreciation of an improved control of their disease [10–12]. There are many different types of alternative and/or complementary therapies, although the botanical drugs are very relevant for the treatment of the intestinal inflammation [13]. This can be mainly related to their safety, since they have been taken from ancient times, in addition to their reputed efficacy, most probably due to the presence of different active components that can concurrently target several pathways or mediators of the inflammatory response. However, most of these uses have an empirical basis, and in consequence, it is necessary to properly evaluate these botanical drugs to consider them as an adequate strategy to treat IBD.

The aim of the present review is to provide scientific arguments that would support the use of medicinal plants as alternative and/or complementary therapy in human IBD. For this purpose, we have focused our attention on those botanical drugs evaluated in human IBD by clinical trials, most of them based on preclinical studies performed in experimental models of colitis. In addition, the mechanisms that may be involved in their intestinal anti-inflammatory effects will be analysed, as well as the main components that can account for the reputed beneficial effects, with a special consideration to polyphenols, including flavonoids, phenylpropanoids, and stilbenes. In fact, these compounds have been well characterized by their antioxidant properties that may prevent the damage caused by reactive oxygen and nitrogen species [26], which have been proposed to be key for the pathogenesis of these intestinal diseases [27].

## 2. Cellular and Molecular Mechanisms Involved in the Inflammatory Response in the Intestine (Figure 1)

**Figure 1:**
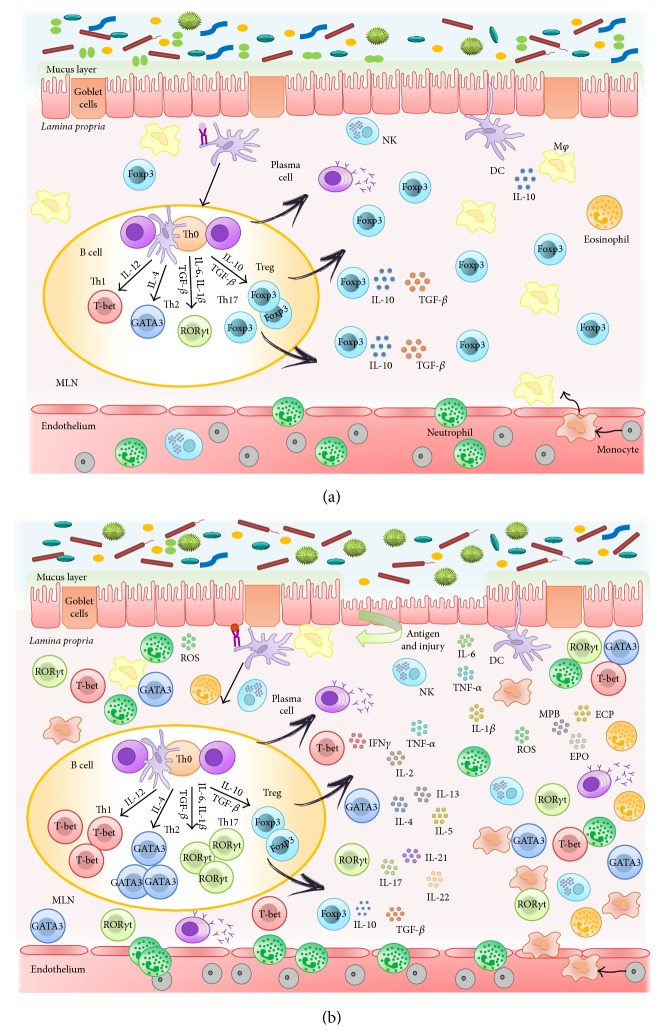
Physiopathology of IBD. (a) The intestine is the largest mucosal surface exposed to the external environment. It constitutes an interface between the host and the luminal contents, which include nutrients and the highest count of resident microbes. Thus, the intestinal immune system meets more antigens than any other part of the body and it must discriminate between invasive organisms and harmless antigens, such as food, proteins, and commensal bacteria, to prevent infections or preserve the homeostasis. This intestinal homeostasis depends on the dynamic interaction between the microbiota, the intestinal epithelial cells, and the resident immune cells, which coordinate a response that keeps the balance between immunity and tolerance. (b) A breakdown of this balance triggers the chronic inflammatory process that characterizes inflammatory bowel disease. There are often several preexisting conditions that lead to the disease: first of all, a genetic susceptibility of the intestinal immune system to distinguish an environmental antigen presented within the gastrointestinal tract; secondly, the contact with the antigen; finally, usually due to an alteration of the permeability, the antigen is presented to the gastrointestinal mucosal immune system through its paracellular passage, which triggers the inflammatory cascade. During early inflammation, luminal antigens activate the different innate immune cells located in the intestine, including natural killer cells, mast cells, neutrophils, macrophages, and dendritic cells, and maintained inflammatory reaction promotes the activation of the adaptive immune response. Abnormally activated effector CD4+ T helper (Th) cells synthesize and release different inflammatory mediators that generate an amplified inflammation that originates from chronic tissue injury and epithelial damage.

The physical barrier of the intestinal epithelium is complemented by a well-evolved mucosal innate immune system, which is poised to defend against pathogenic incursions, and limits inflammatory responses to maintain a state of hyporesponsiveness to commensal bacteria. However, it is also the effector arm that mediates intestinal inflammation. The epithelial-cell layer is comprised of absorptive and secretory cells, goblet cells, and Paneth cells. Goblet cells contribute to the formation of the protective mucus layer [28]. Under physiological conditions,* lamina propria* hosts a large number of different immune cells, including macrophages (M*φ*), dendritic cells (DCs), mast cells, neutrophils, eosinophils, natural killer (NK), NKT cells, and T and B cells. All of these cells coexist in perfect equilibrium that confers tolerance and protection at the same time (Figure 1).

The presence of either pathogenic bacteria or the disruption of the epithelial-cell barrier may result in inflammation and dysregulation of mucosal homeostasis: cells of the innate immunity, such as macrophages and dendritic cells, are specialized in identifying microorganism's molecular patterns by using the pattern recognition receptors (PRR), such as toll-like receptors (TLR) [29]. Moreover, newly recruited monocyte-derived macrophages (and other innate host defense cells) generate cytokines and chemokines to recruit monocytes and other leukocyte populations to contain the inflammation [30]. When innate immunity is no longer able to counteract the pathogen aggression, the adaptive immune response is triggered: DCs migrate to the mesenteric lymph nodes, where they present the antigen to naive T cells and, depending on the factors released by DCs, induce the T cell differentiation [31]. T cells are key players of adaptive immune response that cooperate with other cells and molecules from innate immune system to generate an effective response in order to eliminate the invading pathogens. Upon contact with APCs, naive CD4+ cells have the potential to differentiate into different T helper (Th) subtypes; this process is controlled by the effector cytokines produced by APCs: in presence of IL-12 into Th1 and in presence of IL-4 into Th2, with IL-10 and TGF-*β*, they induce regulatory T cells (iTreg) and with IL-6, IL-1*β*, and TGF-*β*, they induce Th17 cells [32–35]. Each Th subtype exerts specific functions: Th1 cells are essential to eliminate intracellular pathogens, Th2 cells mediate allergic reaction and they confer protection against parasites; and Th17 cells contribute to removing extracellular bacteria and fungi [36, 37]. Then, the interaction between T and B leads to the production of antibodies upon the contact with T cell or DC [38, 39]. Although their main function is the antibody production, B cells can also act as antigen presenting cell and, moreover, they are able to produce cytokines and are involved in maintaining mucosal immune homeostasis [40].

Furthermore, activated leukocytes, such as neutrophils, which are infiltrated in the inflamed mucosa not only generate different proinflammatory cytokines, but also induce oxidative reactions, which markedly alter the redox equilibrium within the gut mucosa, and maintain inflammation by inducing redox-sensitive signalling pathways and transcription factors [41]. Moreover, several inflammatory molecules generate further oxidation products, leading to a self-sustaining and autoamplifying vicious circle, which eventually impairs the gut barrier.

In consequence, and as a result of this complex immune cell activity, IBD, similarly to other inflammatory conditions, is characterized by the involvement of a broad spectrum of inflammatory mediators, including cytokines, chemokines, leukotrienes, and prostaglandins, which actively participate in all the phases of the inflammatory process: initiation, progression, and resolution, when it occurs.

During the active phases of IBD, some chemokines are consistently increased: IL-8 and its receptor, monocyte chemoattractant protein- (MCP-) 1 and MCP-3, and macrophage inflammatory proteins (MIP). Chemokines mediate the recruitment of leucocyte effector populations to the sites of immune reaction and tissue injury since they tightly control leukocyte adhesion and migration across the endothelium, being also able to trigger multiple inflammatory actions including leukocyte activation, granule exocytosis, production of metalloproteinases for matrix degradation, and upregulation of the oxidative burst [42]. Similarly, the upregulated expression of different adhesion molecules in IBD, such as the intercellular adhesion molecule- (ICAM-) 1, the lymphocyte function-associated antigen- (LFA-) 1, the macrophage 1 antigen (Mac-1), the vascular cell adhesion molecule- (VCAM-) 1, the very late antigen- (VLA-) 4, and P- and E-selectins, collaborates in the recruitment of granulocytes and lymphocytes through blood vessels [43].

The roles of cytokines in IBD are very diverse and complex. The fact that these mediators control T-cell differentiation and regulation has made them to be considered as central points of potential intervention to control the inflammatory response. IL-12, IL-18, and IL-23 have a crucial function in Th1 differentiation and chronic activation, whereas other cytokines, such as TNF-*α*, IL-1*β*, and IL-6, augment the inflammatory response by recruiting other cells and enhancing the production of inflammatory mediators [44]. Moreover, IL-23 is induced by pattern recognition receptors (PRRs), whose sustained activation drives chronic intestinal inflammation [45]. Although it was initially linked to the preferential expression of Th17 responses, it can promote a wide range of pathological responses in the intestine, mediated either by T cells or by excessive innate immune activation. IL-23-mediated enhancement of Th1 and Th17 responses is consistent with the increased levels of IFN-*γ*, IL-17, and IL-22 observed in the chronically inflamed intestine [46, 47]. When considering the IL-17 cytokine family, a group of cytokines that includes at least six members, IL-17A, IL-17B, IL-17C, IL-17D, IL-17E (or IL-25), and IL-17F, acts both in vitro and in vivo as potent proinflammatory cytokines [48]. IL-17 can induce the expression of proinflammatory cytokines (like IL-6 and TNF-*α*), chemokines (including keratinocyte chemoattractant (KC), MCP-1, and MIP-2), and matrix metalloproteases, which mediate tissue infiltration and tissue destruction [49], thus playing a key role in human IBD [50]. Finally, it has been reported that an altered production of anti-inflammatory cytokines, including IL-10 and TGF-*β*, can account to the pathogenesis of IBD, because they are considered as key regulators of immunological homeostasis and inflammatory responses in the gut [51, 52].

## 3. Intestinal Anti-inflammatory Effects of Botanical Drugs: Preclinical and Clinical Studies

The growing interest about the potential role that medicinal plant extracts may play in intestinal inflammatory conditions has promoted the development of different clinical studies, thus trying to evaluate their potential efficacy and safety. It is important to note that nowadays most botanical drugs go through a similar rigorous testing as pharmaceutical medicines, in an attempt to avoid inconsistent conclusions. Unfortunately, different factors associated with the design, execution, and interpretation of these clinical trials still make it difficult to easily get clear conclusions with the different strategies to be evaluated against these pathologies [53]. Among these factors, the clinical heterogeneity of both intestinal conditions, UC and particularly CD, can be highlighted, as well as the selection of appropriate therapeutic end points to evaluate the efficacy. In spite of all these concerns, there are positive examples of successful human-controlled trials within the literature of botanical drugs. Although the preclinical studies reporting the beneficial effects of plant extracts on experimental models of colitis are numerous, only a few plant extracts have been used in different clinical assays, which will be described in the present review (Table 1). The main botanical drugs were* Aloe vera*,* Andrographis paniculata*,* Artemisia absinthium*,* Boswellia serrata*,* Cannabis sativa,* and* Curcuma longa*.

### 3.1. *Aloe vera*

**Figure 2:**
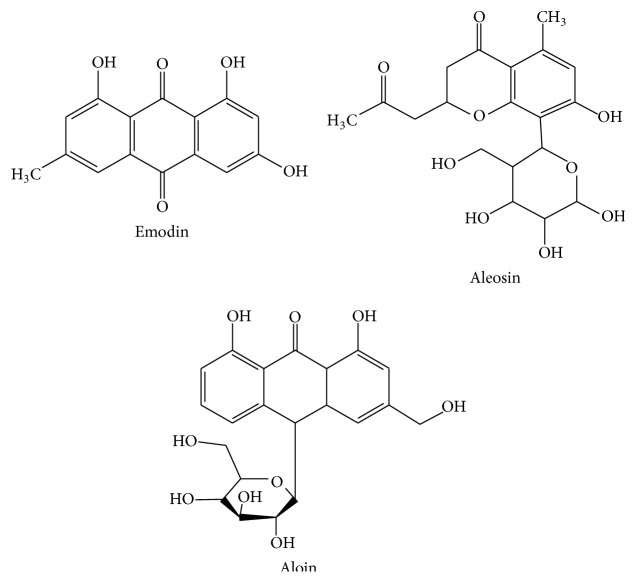
Chemical structures of* Aloe vera* compounds.

*Aloe vera* (Xanthorrhoeaceae) is a tropical plant used in traditional medicine all over the world, mainly for its gel, which is the leaf pulp mucilaginous, aqueous extract. The* Aloe vera* juice has been reported to exert anti-inflammatory activity; therefore, it has been empirically used for the treatment of UC patients [14] (Table 1), being considered as the most popular botanical drug [10]. These beneficial effects have been related to the immunomodulatory properties ascribed to this gel. This was confirmed when the gel was tested in the dextran sulphate sodium (DSS) model of experimental colitis in rats, since it produced an amelioration of the colonic tissue injury induced by DSS, being related to a downregulation of the inflammatory mediators, including cytokines, and attenuation of the immune cell recruitment [54]. Among the different components of the gel, which comprise acetylated mannans, polymannans, anthraquinone C-glycosides, anthrones, anthraquinones (emodin), and lectins, with reputed biological activities, in the same study it was proposed that the chromone aloesin seemed to be essential for the control of the intestinal inflammatory process [54]. It was reported that aloesin, mainly, but also aloin and aloe-emodin (Figure 2) were able to reduce myeloperoxidase (MPO) activity, an enzyme involved in neutrophil activity, an effect that can account in inhibiting the progression of IBD. Moreover, aloesin is a strong inhibitor of leukotriene B_4_ (LTB_4_) that can activate and recruit the inflammatory cells in the injured tissue [55]. Also, these compounds significantly decreased the expression of proinflammatory cytokines, like TNF-*α* and IL-1*β*, in the colonic segment in a dose-dependent manner, being aloesin again the most effective. However, the mechanism through which it exerts this capacity remains unidentified. It is known that this chromone derivative blocks the activation of the NF-*κ*B pathway, thus inhibiting the expression of related proinflammatory genes, including TNF-*α* [56]. Based on the effect observed in retina ganglion cells [57], the anti-inflammatory effect of aloe-emodin and aloin metabolite, in DSS rat colitis, maybe is due to MAP kinase pathways phosphorylation inhibition.

### 3.2. *Andrographis paniculata*

**Figure 3:**
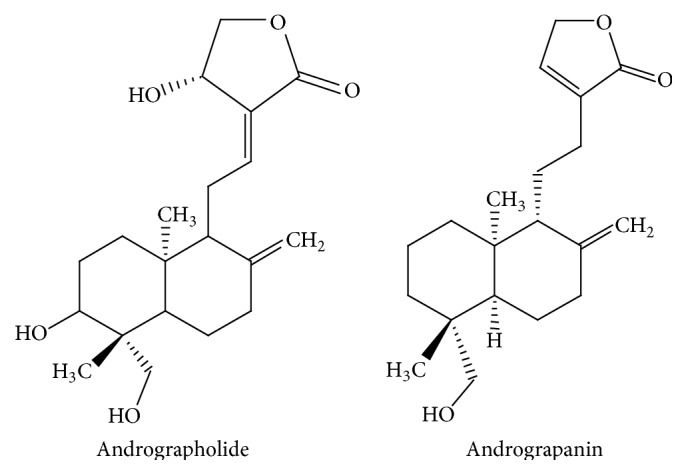
Chemical structures of* Andrographis paniculata* compounds.

*Andrographis paniculata* (Acanthaceae) can be mainly found in India and Sri Lanka, as well as in South and South-Eastern Asia, where its extracts are used as anti-inflammatory remedies [58]. HMPL-004 is a proprietary extract from this plant that has been evaluated for its intestinal anti-inflammatory effects in human trials [15, 16] (Table 1). The phytochemical analyses of the extracts from* Andrographis paniculata* reveal that the main known components are diterpene lactones, principally andrographolide (Figure 3) and its derivatives, which have been reported to exert anti-inflammatory properties through inhibition of the transcription factor NF-*κ*B [59, 60]. Particularly, andrographolide reacts with reduced cysteine 62 of p50 subunit forming a covalent adduct blocking the bond of NF-*κ*B oligonucleotide to nuclear proteins. NF-*κ*B activation promoted the increased expression and synthesis of different proinflammatory mediators involved in the inflammatory response associated with IBD, including chemokines, cytokines, and adhesion molecules, in the different cell types that participate in the altered immune response in these intestinal conditions [61]. For instance, when stimulated-endothelial cells were treated with andrographolide, the reduction of adhesion molecule E-selectin was observed preventing the E-selectin-mediated leukocyte migration [59]. Andrographolide is also able to suppress the expression of inducible nitric oxide synthase (iNOS) as observed in RAW 264.7 cells and, as a consequence, there is a reduction of NO production. This inhibition of NO is due to the blockage of the synthesis and also to the reduction of the stability via a posttranscriptional mechanism [62]. Andrographolide prevents the reactive oxygen species (ROS) production by neutrophils through the modulation of a protein kinase C- (PKC-) pathway. This confers to andrographolide the capacity to downregulate leukocyte integrin Mac-1 (*α*_M_ *β*_2_ CD11bCD18) that it has been reported to be upregulated by ROS. This reduction leads to reducing neutrophil infiltration and transmigration [63]. Besides, andrographolide exerts immunomodulatory properties, most likely affecting the innate immune cells, including macrophages and dendritic cells, but also T cells, by downregulating the production of proinflammatory cytokines [64–66]. It has been observed that andrographolide reduced significantly, in a dose-dependent manner, the IFN-*γ* production in concanavalin A-stimulated murine T cell in vitro, whereas its effects on IL-2 inhibition were partial. Moreover, it can reduce the ERK1/2 phosphorylation that is associated with a reduction of IFN*γ* production [64]. In another study, the ability of andrographolide to interfere with the DCs maturation and with their capacity to present antigen to T cells has been showed. When the DCs were treated with the compound and then were mixed with lymphocytes for allogeneic stimulation, IL-2 release and proliferation were reduced [65]. The extract of* A. paniculata* also contains andrograpanin (Figure 3), a hydrolysate of neoandrographolide (another bicyclic diterpenoid lactone), which also showed anti-inflammatory activity. It was able to reduce the mRNA expression of several genes, including TNF-*α*, IL-16, IL-12p35, and IL-12p40, in a dose-dependent manner. In particular, the reduction of IL-12p35 and IL-12p40 proteins was lower than their mRNA levels, suggesting that andrograpanin applies the major changes at a posttranscriptional level for these two genes [66].

All these studies could justify the inhibitory effects that the extracts of* Andrographis paniculata* may exert on multiple immune cells (DC, macrophages, and T cells) that are implicated in disease development and progression in UC and CD. Supporting this, Michelsen et al. reported the intestinal anti-inflammatory effects of the extract HMPL-004 in a T cell driven experimental model of chronic colitis, by inhibiting the proliferation and/or differentiation of naïve T cells, as well as the Th1/Th17 responses that are activated in intestinal inflammation, being these effects associated with reduced expression of the different proinflammatory cytokines, including TNF*α*, IL-1*β*, IFN*γ*, and IL-22 [67].

### 3.3. *Artemisia absinthium*

*Artemisia absinthium* (Compositae), commonly known as wormwood, is widely distributed all over the world and it is described in different pharmacopoeias, with leaves and stems being used for medicinal purposes. This botanical drug is usually standardised based on its content in the dimeric guaianolides absinthins [68], being considered as a high-quality wormwood when it contains at least 0.2% of absinthin.

**Figure 4:**
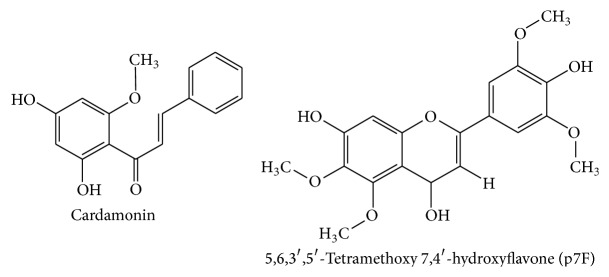
Chemical structures of* Artemisia absinthium* compounds.

Two different clinical trials have reported the beneficial effect of this botanical drug in CD [17, 18] (Table 1). TNF-*α* is considered to play a key role in the pathogenesis of CD, which supports the high efficacy obtained with the biologicals acting as TNF-*α* inhibitors, like infliximab and adalimumab, for severe cases of CD [69]. The results obtained in these clinical trials showed that wormwood administration promoted the clinical improvement of the symptoms in all the patients, whereas no amelioration in the disease was observed in the placebo group. The beneficial effect induced by wormwood was associated with a significant decrease in TNF-*α* serum levels in comparison with those obtained in the placebo group. The suppression of TNF-*α*, as well as of others proinflammatory cytokines like IL-1*β* or IL-6, by wormwood extracts has been reported in vitro as well [70]. Among the components of* Artemisia absinthium*, the flavonoid 5,6,3′,5′-tetramethoxy 7,4′-hydroxyflavone (p7F) (Figure 4) has been isolated that has been shown to exert anti-inflammatory effects. In fact, p7F was able to inhibit the expression of COX-2 and iNOS in LPS-stimulated RAW 264.7 cells. As a consequence of this inhibition, a reduced production of PGE_2_ and NO in the same cells has been observed. Moreover, p7F decreased the activation of NF-*κ*B, induced by LPS, probably through its antioxidant properties [70]. p7F also suppressed the serum levels of TNF-*α* and inhibited NF-*κ*B activation in vivo [70]. Another compound also isolated from this plant, 20,40-dihydroxy-60-methoxychalcone, known as cardamonin (Figure 4), has shown to dose-dependently inhibit NO release and iNOS expression in LPS-stimulated RAW 264.7, as well as NF-*κ*B activation [71]. All these results would support the use of* Artemisia absinthium*, at least as complementary therapy, in IBD.

### 3.4. *Boswellia serrata*

The oleo-gum resin from* Boswellia serrata* (Burseraceae), or Indian frankincense, is a traditional Ayurvedic remedy used to treat inflammatory diseases, including UC. As claimed by a survey performed in Germany, approximately 36% of IBD patients have been administered with* Boswellia serrata* extracts to treat their intestinal condition, reporting positive therapeutic effects [72], and they have been assayed in different clinical trials [19–21] (Table 1).

**Figure 5:**
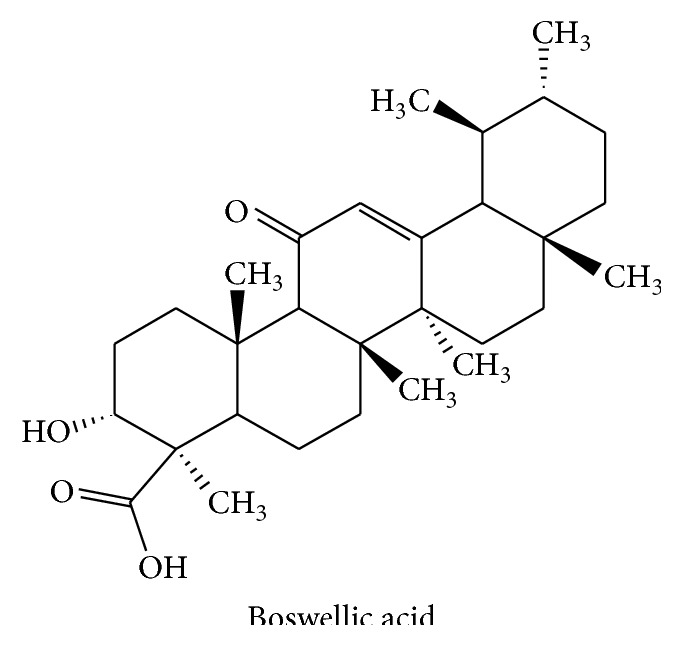
Chemical structure of boswellic acid.

Among the different chemical compounds of this resin, triterpenes are the most abundant (30–60, depending on its origin), with boswellic acids being the major constituents (Figure 5), which are thought to largely contribute to the pharmacological activities such as anti-inflammatory and antiarthritic effects ascribed to this crude drug [73]. In vivo experiments performed in the acetic acid model of rat colitis have proposed that the antioxidant properties of the extracts from* Boswellia serrata* may also account for their intestinal anti-inflammatory effect [74, 75]. It has been shown that* Boswellia serrata* extract reduced the lipid peroxidation, while it increased the levels of superoxide dismutase (SOD), thus ameliorating the oxidative stress associated with intestinal inflammation.

Additional mechanisms can also account for their beneficial effects. Thus, in vitro experiments have shown that these compounds decreased the leukotriene formation by blocking the 5-lipoxygenase pathway which can account in the beneficial effect showed by this botanical drug since leukotrienes have been clearly involved in the pathogenesis of IBD [76]. Similarly, the anti-inflammatory effects of boswellic acids seem to involve the inhibition of different cellular pathways including those related to the transcription factor NF-*κ*B activation, which has been described to induce the expression and/or to activate the function of many proinflammatory cytokines, like TNF*α*, IL-1*β*, and IL-6, that are crucial for the development and the maintenance of intestinal inflammation [77, 78]. Surprisingly, contradictory results have been reported when the effectiveness of* Boswellia* extracts was assayed in DSS- or trinitrobenzene sulfonic acid- (TNBS-) induced experimental models, since no efficacy was demonstrated in ameliorating colitis in these models [79]. Furthermore, in the same study, the ability of different boswellic acids to enhance the basal and the IL-1*β*-stimulated NF-*κ*B activity in intestinal epithelial cells was demonstrated, as well as the potential hepatotoxic effect of* Boswellia*, claiming for an special attention with the use of this botanical drug in IBD and other inflammatory disorders [79]. On the contrary, a semisynthetic form of acetyl-11-keto-*β*-boswellic acid lessened the disease activity index in DSS colitis, and it managed to diminish the recruitment of leucocytes and platelets, maybe due to its ability to prevent P-selectin upregulation, thus protecting colonic mucosa from DSS insult. The beneficial effects showed by this derivative were similar to those obtained with the standard corticosteroid dexamethasone [80].

### 3.5. *Cannabis sativa*

*Cannabis sativa* (Cannabaceae) has been long employed for the treatment of different diseases, especially for chronic pain and different neurological conditions [81, 82]. Moreover, this botanical drug has treated different gastrointestinal conditions including anorexia, emesis, abdominal pain, diarrhea, and diabetic gastroparesis [83]. It has been reported to contain over 60 different cannabinoid compounds, which are responsible for the biological activities reported for* Cannabis sativa* [84]. In addition, experimental evidence suggests that the endogenous cannabinoid system is involved in most of the major immune events, including those located in the gastrointestinal tract [85, 86]. For this reason, it was proposed that the activation of this system by cannabinoids might have a therapeutic role in human IBD [87]. However, and although its use is common in IBD patients, there are few controlled studies that evaluate the exact role of cannabis in IBD [22, 23, 88] (Table 1).

**Figure 6:**
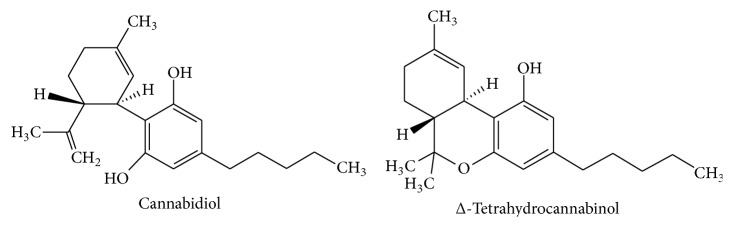
Chemical structures of* Cannabis sativa* compounds.

The mechanisms involved in the intestinal anti-inflammatory effects of cannabis can be related to the capacity of cannabinoids to downregulate the production and release of different proinflammatory mediators including TNF*α*, IL-1*β*, and nitric oxide, thus restoring the altered immune response that occurs in IBD [89]. Most probably, these effects would be related to cannabinoid receptors type 1 (CB1) activation that mediates essential protective signals and counteracts proinflammatory pathways, since it has been reported that the severity of two different experimental models of colitis, induced by the intrarectal infusion of 2,4-dinitrobenzene sulfonic acid (DNBS) or by oral administration of DSS, are higher in CB1-deficient mice (CB1(−/−)) than in wild-type [90]. Lack of CB1 receptors rendered mice more sensitive to inflammatory insults, indicating a protective role of the CB1 receptors during inflammation induction. Consistently, the administration of a specific CB1 antagonist to these wild-type mice before colitis induction resulted in a similar degree of intestinal damage to that seen in CB1(−/−) mice, whereas the administration of a cannabinoid receptor agonist protected from DNBS-induced colitis in mice [90]. Supporting these observations, both Δ-tetrahydrocannabinol (THC) and cannabidiol (CBD) (Figure 6), two of the main components of cannabis, were able to exert beneficial effects in the TNBS model of acute colitis in rats, with a similar efficacy to that shown by sulphasalazine, which was used as a positive control [91]. Particularly, treatment with THC and combined treatment with CBD was able to reduce macroscopic damage score. Both alone and in combination, THC and CBD reduced the MPO activity similarly to treatment with sulphasalazine [91].

Unfortunately, the main concern with the use of cannabis in IBD can be derived from the indiscriminate binding of the cannabinoids to the receptors in the central nervous system, which may result in serious side effects including dizziness, dry mouth, nausea, fatigue, somnolence, euphoria, vomiting, disorientation, drowsiness, confusion, loss of balance, and even hallucination [92]. It is evident that the manipulation of the endocannabinoid system can be helpful for the management of human IBD; however, additional research needs to be carried out to consider cannabis (and cannabinoids) as a suitable medicine in these gastrointestinal conditions. Maybe, the use of specific cannabinoids can constitute an attractive alternative. This may be the case of cannabidiol, which possesses anti-inflammatory and immunomodulatory properties, as demonstrated in experimental models of rodent colitis, although it lacks of any cognitive or psychoactive effects [93].

### 3.6. *Curcuma longa*

**Figure 7:**
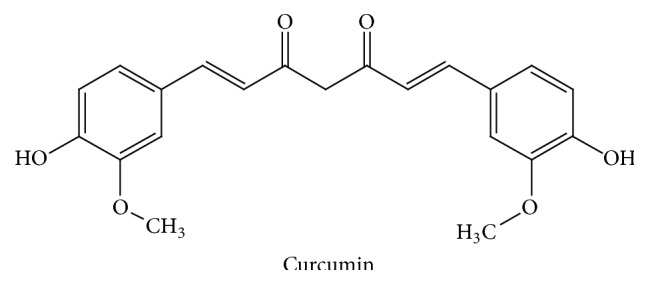
Chemical structure of curcumin.

*Curcuma longa* (Zingiberaceae) is commonly known as Turmeric. It is an Indian spice obtained from the rhizomes of the plant, which has been long used in Ayurvedic medicine for the handling of different inflammatory diseases [94]. Curcumin (Figure 7) is the main active component of turmeric that also provides its vibrant yellow colour [95]. Numerous pharmacological activities have been reported for curcumin, including antioxidant, antimicrobial, anticancer, and anti-inflammatory properties. Regarding the latter, curcumin has been suggested to exert positive effects in IBD, although just a few clinical studies have shown the power of curcumin to prevent and/or ameliorate this condition.

Clinical studies showed that the concurrent administration of curcumin and standard drugs improved their efficacy [24, 25] (Table 1) and seemed to be well tolerated after a prolonged administration, which can be considered as a secure medication for maintaining remission and preventing relapse [96].

The preclinical studies performed with curcumin have shown its efficacy in different experimental models of colitis, either chemically induced or in knockout mice, and following different protocols of administration, that is, preventative or curative [97–102]. Besides, all these studies have rendered important information about the possible mechanisms implicated in these beneficial effects. Among these, the antioxidant properties of curcumin, derived from its ability to scavenge free radicals, may play a prominent role, given the oxidative stress that characterizes these intestinal conditions [103]. In addition, curcumin can also modify multiple signaling pathways, especially the kinases MAPK and ERK, thus affecting the expression of different proteins implicated in the intestinal inflammatory cascade, like MPO, COX-2, iNOS, or LOX [104]. Additionally, a modulation of the altered immune response has been proposed to occur after curcumin administration in experimental colitis, by attenuating the production of proinflammatory cytokines, like TNF*α*, IL-1*β*, IL-12, or IFN*γ*, but increasing of the expression of anti-inflammatory cytokines [98, 101]. Other mechanisms involved in the beneficial effects of curcumin can be related to an inhibitory effect of NF-*κ*B activity [105]. In fact, it has been reported that elevated levels of NF-*κ*B in IBD proportionately amplified the production of inflammatory cytokines and resulted in mucosal damage, which in turn can upregulate the production of this transcription factor, promoting a recurring feedback loop of inflammation [106]. In consequence, the ability of curcumin to modulate NF-*κ*B activation may prevent the inflammatory response, thus preventing colonic mucosa damage.

In conclusion, the results reviewed show that botanical drugs might prompt clinical remission and a clinical response in IBD patients. In particular, botanical drugs significantly induce clinical remission in CD patients and clinical response in UC patients; however, there was not a significant induction of clinical remission in UC patients and an obvious clinical response in CD patients. The results of subanalyses taking into account the plant type demonstrate that only* Artemisia absinthium* and* Boswellia serrata* were able to induce clinical remission, while* Aloe vera* induced a clinical response. However, none of the plants confirmed endoscopic or histological efficacy. On the other hand, none of the plants produced any adverse effects in comparison with placebo. So, although some of the botanical drugs may have clinical efficacy in patients with IBD, there is too limited evidence to make any strong conclusions. However, botanical drugs could still be safer than synthetic drugs, despite the fact that they are not completely devoid of risk.

## 4. Perspectives

The potential additional benefits of botanical drugs could be that patients accept them, besides their efficacy, acceptable safety, and comparatively low cost. Patients all over the world seem to be prone to use botanical drugs, and their efficacy has been now evaluated in multitude of clinical trials in the management of UC. However, the evidences are still partial, intricate, and puzzling and unquestionably related to both benefits and side effects.

First of all, there should be a deeper knowledge of their composition, the active compounds that are responsible of their properties, and their side effects or toxicity. It is also important to control the harvest of the right plants, their quality, and the later processing to ensure the stability of the active components. Therefore, there is a need for a regulation that establishes the quality standards of the botanical drugs that are sold.

Secondly, further controlled clinical trials, with larger number of patients, are required to evaluate the potential efficacy and toxicity of botanical drugs in the treatment of UC. In this regard, it is also important to let the doctors know any evidence so they can prescribe these botanical drugs with the maximal guarantee.

## Figures and Tables

**Table 1:** Clinical trials of botanical drugs in patients with inflammatory bowel disease.

Herbal preparation	Study design	Number of patients	IBD type	Dose	Comparator	Frequency	Endpoint	Reference
*Aloe vera*	Randomized, double-blind controlled study	44	UC	100 mL twice/day	Placebo	4 weeks	*Aloe vera* produced a significantly better clinical response than in those receiving placebo. The Simple Clinical Colitis Activity Index and histological scores decreased significantly during treatment with *Aloe vera* but not with placebo	[14]

*Andrographis paniculata * (HMPL-004)	Randomized, double-blind multicentre study	120	UC	1.2 g/day	Mesalazine (4.5 mg/day)	8 weeks	There were no significant differences between the two treated groups when considering the clinical efficacy rates or the safety profile	[15]
	Randomized, double-blind placebo-controlled study	224	UC	1.2 g/day and 1.8 g/day	Placebo	8 weeks	Patients treated with the extract, mainly at the highest doses, were more likely to achieve clinical response than those receiving placebo, whereas the incidence of adverse events was similar among groups, although the occurrence of rash was higher in the HMPL-004 extract groups	[16]

*Artemisia absinthium*	Randomized, double-blind multicentre study	40	CD	3 × 500 mg/day	Placebo	10 weeks	After 8 weeks of treatment with wormwood, there was almost complete remission of symptoms in 65% of the patients, whereas no beneficial effect was observed in those receiving the placebo	[17]
	Randomized, double-blind multicentre study	20	CD	3 × 750 mg/day (in addition to standard therapy)	Standard therapy + placebo	6 weeks	Wormwood administration promoted the clinical improvement of the symptoms in all the patients. The beneficial effect was associated with a significant decrease in TNF*α* serum levels in comparison with those obtained in the placebo group, where no amelioration in the disease was observed	[18]

*Boswellia serrata* (Gum resin)	—	?	UC	750 mg (3 × 250 mg)	Sulfasalazine 3 g (3 × 1 g)	6 weeks	All parameters tested improved after treatment with *Boswellia serrata* gum resin, with the results being similar compared to controls: 82% out of treated patients went into remission; in case of sulfasalazine remission rate was 75%	[19]
(Gum resin)	—	30	UC?	900 mg (3 × 300 mg)	Sulfasalazine 3 g (3 × 1 g)	6 weeks	Patients showed an improvement in several parameters: stool properties, histopathology, and scanning electron microscopy, besides haemoglobin, serum iron, calcium, phosphorus, proteins, total leukocytes, and eosinophils. The remission was higher in patients treated with *Boswellia serrata*	[20]
(Boswelan)	Randomized, double-blind, multicentre placebo-controlled study	82	CD	2.4 g/day	Placebo	12 months (52 weeks)	Boswelan showed a safety profile during the long-term therapy but the results obtained did not show a higher efficacy when compared with placebo	[21]

*Cannabis sativa*	Retrospective observation study	30	CD	—	—	—	Cannabis administration was associated with an improvement in disease activity and a reduction in the need of other medications, as well as a reduced risk of surgery	[22]
	Prospective Placebo-controlled study	21	CD	2 cigarettes containing 115 mg of THC/day	Placebo	8 weeks	A significant amelioration of the CD activity index has been reported in the majority of the subjects after cannabis treatment in comparison with placebo administration; in fact, complete remission was achieved in half of the subjects in the cannabis group, whereas it only occurred in 10% of the placebo group patients	[23]

*Curcuma longa*	Open-label pilot study Open-label pilot study	5 5	UC CD	1.100 g/day (550 mg × 2) for 1 month, then 1.650 g/day (550 mg × 3) for 1 month and 1.080 g/day (360 mg × 3) for 1 month, and then 1.440 g/day for two months	— —	2 months 3 months	The results from this study revealed that the treatment of these patients with curcumin for two months resulted in an overall improvement in all the patients, as evidenced by amelioration of the serological parameters evaluated (erythrocyte sedimentation rate and C-reactive protein) as well as the disease activity index followed, together with a reduction in the dose of medication, or even suppression. In the CD group, all patients also reported fewer bowel movements, less diarrhoea, and less abdominal pain and cramping	[24]
	Randomized, double-blind multicentre placebo-controlled study	89	UC	2 g/day plus sulfasalazine or mesalazine	Placebo plus sulfasalazine or mesalazine	6 months	The relapse rate was significantly higher in the placebo group, receiving only the aminosalicylate (20.5%), than in the curcumin-treated cohorts (4.7%). During the period of the study, a marked reduction of the disease-associated clinical activity index and the endoscopic index scores was reported	[25]
